# Editorial: Yeast Differentiation: From Cell-to-Cell Heterogeneity to Replicative Aging and Regulated Cell Death

**DOI:** 10.3389/fcell.2021.823447

**Published:** 2022-01-04

**Authors:** J. Marie Hardwick, Dmitry Knorre, Zdena Palkova, Joris Winderickx

**Affiliations:** ^1^ Department of Molecular Microbiology and Immunology, Johns Hopkins University Bloomberg School of Public Health, Baltimore, MD, United States; ^2^ Belozersy Institute of Physico-Chemical Biology, Lomonosov Moscow State University, Moscow, Russia; ^3^ Faculty of Bioengineering and Bioinformatics, Lomonosov Moscow State University, Moscow, Russia; ^4^ Faculty of Science, BIOCEV, Charles University, Prague, Czechia; ^5^ Department of Biology, Functional Biology, KU Leuven, Heverlee, Belgium

**Keywords:** yeast, programmed cell death, biofilm, heterogeneity, differentiation

Despite being unicellular, yeasts are still capable of differentiation within their natural community. Yeasts grow as a colony or biofilm originating from a single cell or group of cells. As the amount of nutrients decreases, some cells in the population begin to differentiate. Thus a single colony or biofilm shows significant internal heterogeneity. This heterogeneity occurs for a variety of reasons, including amplified transcriptional noise, asymmetric distribution of damaged/aged cells, and microenvironmental differences that can signal to further drive differentiation within a community. Genetically highly related yeast cells within a population can exhibit significantly different growth and death behaviors explained by genetic and non-genetic mechanisms (Teng et al., 2013; Dhar et al., 2019). Further elaborating on this topic, Arabaciyan et al. describes a new experimental pipeline to assess the correlation between the concentration of a protein in a single cell with the growth rate of a microcolony originating from it, an approach that will accelerate studies of “noisy” promoters as likely drivers of some types of yeast colony differentiation.

Spatial positioning within the community also causes subpopulations of yeast to express different sets of genes, take on different roles in the community, and interact with each other differently (Palková and Váchová, 2021). For instance, biofilms of pathogenic yeasts are highly resistant to antifungals, in part because these compounds cannot reach the inner layers of the biofilm, where fungi are protected by pathogen-derived extracellular matrix (ECM). This inspired Tits et al. to explore the antibiofilm effects of a quaternary ammonium compound (domiphen bromide) when combined with a fungicidal azole. They observed that domiphen bromide has a profound impact on the intracellular distribution of a fluorescently labeled azole (FKD), enhancing the levels of oxidative stress and fungal cell death in biofilms. In another study, Chen et al. performed a genetic screen on *Candida glabrata* to identify genes contributing to biofilm formation. They found that deletion of the SNARE protein homologue Syn8 reduces cell adhesion during biofilm formation and sensitizes *C. glabrata* to the antifungal agent hygromycin B, a protein synthesis inhibitor.

It is well known that a mother yeast cell stops dividing and dies after budding off a series of daughter cells, a phenomenon referred to as replicative aging and is thought to contribute to early stages of yeast colony differentiation. Mitochondrial metabolism and chromatin silencing define the changes that precede the death of the ageing mother cell (Li et al., 2020). Using the fungal model *Podospora anserina*, Heinz et al. now show that aging is not only connected to energy metabolism but also to the mating type, as different mutations in the mating-type linked protein RMP1 can shorten or lengthen life span. Mitochondrial versus cytosolic localization is known to depend on developmental stages of *P. anserina* (Contamine et al., 2004). As such, the study of Heinz et al. unveils an interesting connection between mitochondria, developmental processes and lifespan control in this fungal model organism. In addition, Kovacs et al. demonstrated that lipid droplets sustain a potent detoxification mechanism and that increasing their number slows down aging and extends the longevity of *S. cerevisiae* cells.

In metazoa, programmed cell death (PCD) enables embryonic development and tissue homeostasis, leading to the long-standing assumption that PCD arose with multicellularity. However, an emerging consensus suggests a reverse order, where PCD mechanisms likely arose in unicellular species and was required for multicellularity (Durand, 2020). Although fungal genomes encode homologues of a few mammalian proteins involved in PCD, the biological role of these proteins and their contribution to yeast PCD cannot be inferred based on sequence homology alone. A good example is cytochrome *c*, which shuttles electrons between complexes III and IV in the respiratory chain, and upon activation of the PCD cascade, can be released from mitochondria into the cytosol and trigger apoptotic caspase activation in mammalian cells (Yin and O’Neill, 2021). However, forced tethering of cytochrome *c* to the yeast mitochondrial inner membrane via a transmembrane tag does not prevent oxidative stress-induced cell death (Toth et al., 2020), suggesting that other mechanisms are at play in the regulation of yeast cell death. In a comprehensive review, Chaves et al. collected and critically analyzed the mechanisms of acetic acid-induced regulated cell death in yeast that have been established so far.

Although it is still a matter of debate whether the biochemical processes in dying yeast cells reflect adaptive evolution, several special scenarios indicate that this may well be the case. Examples are the destruction of spores during meiosis under the conditions of carbon source deficiency (Eastwood et al., 2012) or the propagation of yeast multicellular aggregates or propagules in suspension cultures (Ratcliff et al., 2012). These findings exemplify that by inducing the death of a subset of cells, both metazoa and yeast accomplish different goals. Thus, the absence of obvious mammalian apoptosis homologs in yeast may not be surprising, and several manifestations and conditions accompanying PCD in yeast may be specific to fungi. We hope that the articles in this issue as well as future studies will help to unveil new mechanisms mediating yeast differentiation, including the processes underlying aging and cell death.
